# Socio-economic Conditions & Determinants of Income: A Tell of Informal Workers of Urban Odisha, India

**DOI:** 10.12688/f1000research.168208.1

**Published:** 2026-02-13

**Authors:** Suvendu Barik, Nishi Kanta Mishra, Monika Saxena

**Affiliations:** 1 School of Economics & Commerce, Kalinga Institute of Industrial Technology, Bhubaneswar, Odisha, 751024, India; 2 School of Liberal Studies, Kalinga Institute of Industrial Technology, Bhubaneswar, Odisha, 751024, India; 3 School of Management, Bennett University, Greater Noida, Uttar Pradesh, 201310, India

**Keywords:** Regional Migration, Informal Labour Market, Determinants of Income, Public Policy for Sustainable Development Goals

## Abstract

**Background:**

Informal employment and its impact on economic growth have been receiving greater attention, but less is known about their socioeconomic conditions and determinants of income in India. This column attempts to uncover the determinants of informal income and socioeconomic conditions with respect to migration status.

**Methods:**

This study was based on primary investigation. Hence the data collection was performed on multi-stage stratified sampling method. The primary data has been collected through a structured questionnaire for the informal sector workers in Cuttack city of Odisha. The data analysis was completed by ANOVA and multiple regression analysis.

**Results:**

The income of migrant workers is more than the non-migrant workers due to their nature of work and pattern of payment, whereas the non-migrant workers are engaged in household services with lower payment. The important socio economic determinants such as category of land they lived, gender, various age groups of household head, migration status, availability of off-season support, type of employment, social group, religion, principal sectors, number of dependency, and members of the union are responsible for lower income of the informal workers.

**Conclusion:**

For sustainable and equitable improvements in basic facilities such as housing, electricity, health, vocational education, social security and employment opportunity should be facilitated by the policy makers. In order to achieve these objectives, this study emphasized more on the mobile-education-services, MGNREGS programs and the MSMEs sector by which Sustainable Development Goals 1,2,3,4,6,8,9,12, and 17 can simultaneously be achieved.

## Introduction

Informal sector employment ^1^ and its impact on economic growth have received greater attention not only in India but also in all underdeveloped and developing countries in the world ( Banerjee, 1983; Breman, 1996; Datta, 2016; Himanshu, 2019; Jha and Lahoti, 2021; Barik, 2024). In India, it is about 91 percent of the total labor force (475 million) in the informal sector, more than 90 percent in the unorganized sector, and about 91 percent additional laborers were engaged in the unorganized informal sector only (Economic Survey Report 2021-22; Mehrotra, 2022). It is in excess of 92 percent, both in the informal and formal sectors, working as self-employed or casual laborers, and approximately 50 percent of the national income appears in this informal sector. Similarly, the NSSO 73 ^rd^ round report (2015-2016) analyzed that major states such as Uttar Pradesh, West Bengal, Tamil Nadu, Maharashtra, and Karnataka occupy all most 50 percent of the workers. The state such as Himachal Pradesh was the lowest contributor (0.58%) to the total workforce, and Uttar Pradesh had the highest (15%) in India. However, the state of Odisha has been occupied with the 13 ^th^ position (3% of workers) in engaging in the unincorporated non-agricultural sector in India, except for the construction sector. It accounts for 4.74 percent of workers from the rural areas and 1.57 percent in urban areas, and 98.74 percent and 98.31 percent, respectively, of the total workforce in India. The condition of informal workers in Odisha is diverse in nature, because of the struggle for the survival of the mass poor, lack of suitable job, non-subsistence level of wage rate, and non-sustainability of the work or due to the ‘vicious circle of poverty’.

Therefore, in informal sector employment, the income level and determinants of income are of paramount importance. As a result, this study conducted an empirical investigation to understand the different difficulties related to informal sector workers (ISWs) in Odisha, specifically Cuttack. The CTC (Cuttack City) was selected as the sample area, as it is the 1 ^st^ capital city and also concentrate the 2 ^nd^ highest informal sectors employment (after Bhubaneswar) in Odisha. However, the main purpose of this research is to discover the main determinants of the income of vulnerable ISWs working in the CTC of Odisha. The rest of the study is organized as follows: review of the literatures ( section-2), methodology and data ( section-3), empirical analysis and discussions ( section-4), and finally, conclusions and policy recommendations ( section-5).

## Review of literature

In this paper, the literature review is followed by international-level studies (studies made other than India), national-level studies (study at the national level excluding Odisha), and regional-level studies (specifically in Odisha) to understand the causes and consequences of income variation among workers.

The literature review begins with the ‘Blinder-Oaxaca Decomposition Theorem, 1973. According to the author, the main causes of wage discrimination among white, black, male, and female workers in the USA are differences in age-wage profiles, unequal income distribution, union membership, occupational status, educational discrimination, and job experience. On the other hand, the author noted that the major causes of male-female wage discrimination in the USA include variations in tradition, culture, and overt discrimination within the same occupation ( Meher and Dash, 2014). In this study, the existence of racial income differences (minorities and employment discrimination), which play a crucial role in the income gap between black and white workers, as not considered. Lower income of the black workers is due to very less investment on the human capital, ‘taste for discrimination’ for the black workers and the acquisition of market valued characteristics than the white workers. Similarly, in France, the pay gap among workers is due to gender bias, demand for skilled workers, and non-monetary gains such as job protection in the civil sector ( Odisha Economic Survey, 2020-21 and Tandon, 2022). In Europe, disparities in working conditions, occupational exposure, gender, health conditions, immigration status, and employment arrangements are major contributors to the pay disparity between workers (both non-migrants and migrants). According to the IMF (2015) and ILO(2015 and 2021) income inequality sharply worldwide due to the inequality of opportunity for obtaining education, occupational choices reducing skills, weaker social mobility, and workplace inequalities. Income inequality is increasing in emerging economies of G-20 (i.e., Indonesia and China) and decreasing in developing countries (i.e., Brazil and Argentina) due to social and political cohesion, less sustained economic growth, and lower consumption, according to the ILO, IMF, OECD and WBG (2015). Similarly, according to the ILO report (2021), income inequality increases sharply under COVID-19 due to a decrease in relative levels of income, quality of employment opportunities, and inter-sectional division among workforce groups (i.e., on the basis of race and ethnicity, education, age, migration status, formal and informal workers, etc.). On the one hand, Blinder (1973) found that the differences in the age-wage-profile, unequal income distribution, membership in the union, occupational status, educational discrimination and job experience are the main reasons for wage discrimination among white-black and male-female workers in the USA. On the other hand, Oaxaca (1973) observed that differences in tradition, culture, and overt discrimination within the same occupation are important reasons for the male-female wage discrimination in the USA. According to Loury (1977), racial income differences (minorities and employment discrimination) which play a vital role in the income difference between black and the white workers have not been taken into consideration. Lower income of the black workers is due to very less investment on the human capital, ‘taste for discrimination’ for the black workers and the acquisition of market valued characteristics than the white workers. Similarly, in France, the pay gap among workers is due to gender bias, demand for skilled workers, and non-monetary gains such as job protection in the civil sector. However, in Europe, the important reason for the income variation between workers (both non-migrant and migrant) is the difference in working conditions, occupational exposure, gender, health condition, migration status, and employment arrangements ( Perez et al., 2012).

In recent years, income inequality has increased worldwide due to the inequality of opportunities for obtaining education, occupational choices reducing skills, weaker social mobility, and workplace inequalities. Income inequality is increasing in emerging economies of G-20 (i.e., Indonesia and China) and decreasing in developing countries (i.e., Brazil and Argentina) due to social and political cohesion, less sustained economic growth, and lower consumption, according to the ILO, IMF, OECD and WBG (2015). Similarly, according to the ILO report (2021), income inequality increases sharply under COVID-19 due to a decrease in relative levels of income, quality of employment opportunities, and inter-sectional division among workforce groups (i.e., on the basis of race and ethnicity, education, age, migration status, formal and informal workers, etc.). Some macroeconomic determinants of income inequality among different income groups have also been identified from panel data by Basumatary et al. (2024). According to the different income-group countries from 1996 to 2019, the main determinants of low-income countries with a positive impact on income inequality are the HDI, civil liberty, and governance. In lower-middle-income countries, there is a positive association between population growth, globalization, governance, and income inequality. In upper-middle-income countries, economic and population growth, civil liberty, and governance are positively related to income inequality. However, in high-income countries, economic growth, population growth, gender equality, and natural resources have a positive impact on income inequality ( Basumatary et al., 2024). A primary work-based study on female informal workers in Hosanna City, Ethiopia, found that socio-economic advancement is also largely influenced byage, work experience, income, savings, and migration ( Lemma and Sharma, 2025).

In the case of India, Banerjee (1983) explained that a potential migrant has the ability to know his/her real-income differentials at the time of urban informal sector employment or unemployment compared to the formal sector. There is a huge income gap between informal and formal sector employment due to the high wage rate of formal workers, a subsistence level or lower than the subsistent level wage rate of informal workers, lack of human capital, and institutional barriers. Owing to the lack of trade unions, minimum wage laws, unemployment insurance, and welfare benefits in traditional economies, poor workers have a poorer standard of life, which contributes to the income gap. Some of the studies in India observed that the possibility of an informal individual being poor is less in a large city than in any other rural area; in case of the migrant people, it is the higher caste category than the lower caste people. This is due to the existence of different levels of monthly per capita income and consumption in urban and rural areas (due to the impact of urbanization and globalization) and the wage disadvantages of non-migrants in rural areas ( Breman, 1996; Dabla-Norris et al., 2015). The difference exists among the higher and lower caste categories of people because of the changes in the real wages over time among the male and female workers and the work transformation from agricultural to the non-agricultural work. Due to the overrepresentation of female migrant workers in casual labor, the author noted that migrant workers have lower salary rates than non-migrant workers, although migrants have wage advantages for male workers in regular jobs. According to the study, there is a salary disparity between state and private mining in India (Samanta et. al., 2022). According to them, workers working in the public sector earn higher wages than those in the private sector, and the wage gap is higher at the bottom and lower at the top owing to unequal wage distribution. Similarly, inter-industry wage differentials have also been observed in India due to the absence of convergence and persistence among the manufacturing sector, for which the same skilled laborers receive different wages in different manufacturing industries. According to Padhi et al. (2019), the main reasons for gender-based wage discrimination in India are mostly non-monetary factors such as defeminisation, casualization, and informalization. Other non-monetary factors such as social problems, education, health, and social protection play a vital role in increasing inequality in India ( Himanshu, 2019; Munshi and Singh, 2024). Another important factor that also plays a crucial role in increasing the income differences between the low-income households is the COVID-19 pandemic ( Jha and Lahoti, 2021). This pandemic not only worsens the financial situation of the nation but also breaks down the backbone of the financial support of many households. However, due to the significant growing technological progress and excess supply of workers, the capital share is increasing significantly at the expense of labor share, but technological progress can increase labor share by mounting suitable employment opportunities ( Ozdemir, 2023).

In the case of out-migrants in Odisha, the determinants of remittance sending are different due to their social, economic, and demographic characteristics, and economic growth in some sectors ( Odisha Economic Survey, 2020-21). According to Meher and Dash, not all out-migrants are able to send the same remittances to their native place of Odisha due to age, sex, place of residence and social group. However, Sahoo and Paltasingh explained that income inequality rises because of the significant growth in some sectors, specifically in the tertiary sectors after the post-reform period. However, inequality in income arises mostly due to the outbreak of COVID-19, and as unemployment increases at a high time, a wage-cut policy was adopted by some of the IT sectors, leading to poor education, reduction in government expenditure except health, and negative economic growth in different sectors differently, etc. ( Odisha Economic Survey, 2020-21). Similarly, due to various socio-economic factors, inequality in income and expenditure is also increasing among non-migrant and migrant informal workers in the rural and urban areas of the country ( Barik and Baig, 2022; Barik, 2024). Thus, it is logical that very few studies have been conducted on the informal sector, informal employment, and informal income at the regional level. It is also important to study the dynamics of informal workers and their determinants of income, specifically among migrant and non-migrant ISWs. Therefore, this paper focuses on the empirical study of the Cuttack (CTC) city of Odisha, as the CTC is the oldest, the 1 ^st^ capital city and 2 ^nd^ largest ISWs.

## Methods

The present case study is based purely on a household-level primary survey conducted during March-May 2025 in the CTC of Odisha. In this study, the number of household heads (HHds) was 470 (10 percent criteria of the total slum population was applied), which included both non-migrant (NMs) and migrant (Ms) slum-dwellers, specifically informal sector workers (ISWs). HHds are selected based on the strength of the respondent’s occupation (self-employed, wage-employed, and casual laborers), migration status (whether migrated or non-migrated), and keeping in mind the municipality classification (CTC has three Constituencies; Barabati, Chaudwar and Sadar). Thus, the present study was followed by a multi-stage stratified sampling method for collecting relevant data from the ISWs of the CTC, Odisha. However, before collecting the information from the informal respondents, secondary information was also taken into consideration that is from the Bikash Bhavan CMC (Cuttack Municipality Corporation).

**Figure 1.**
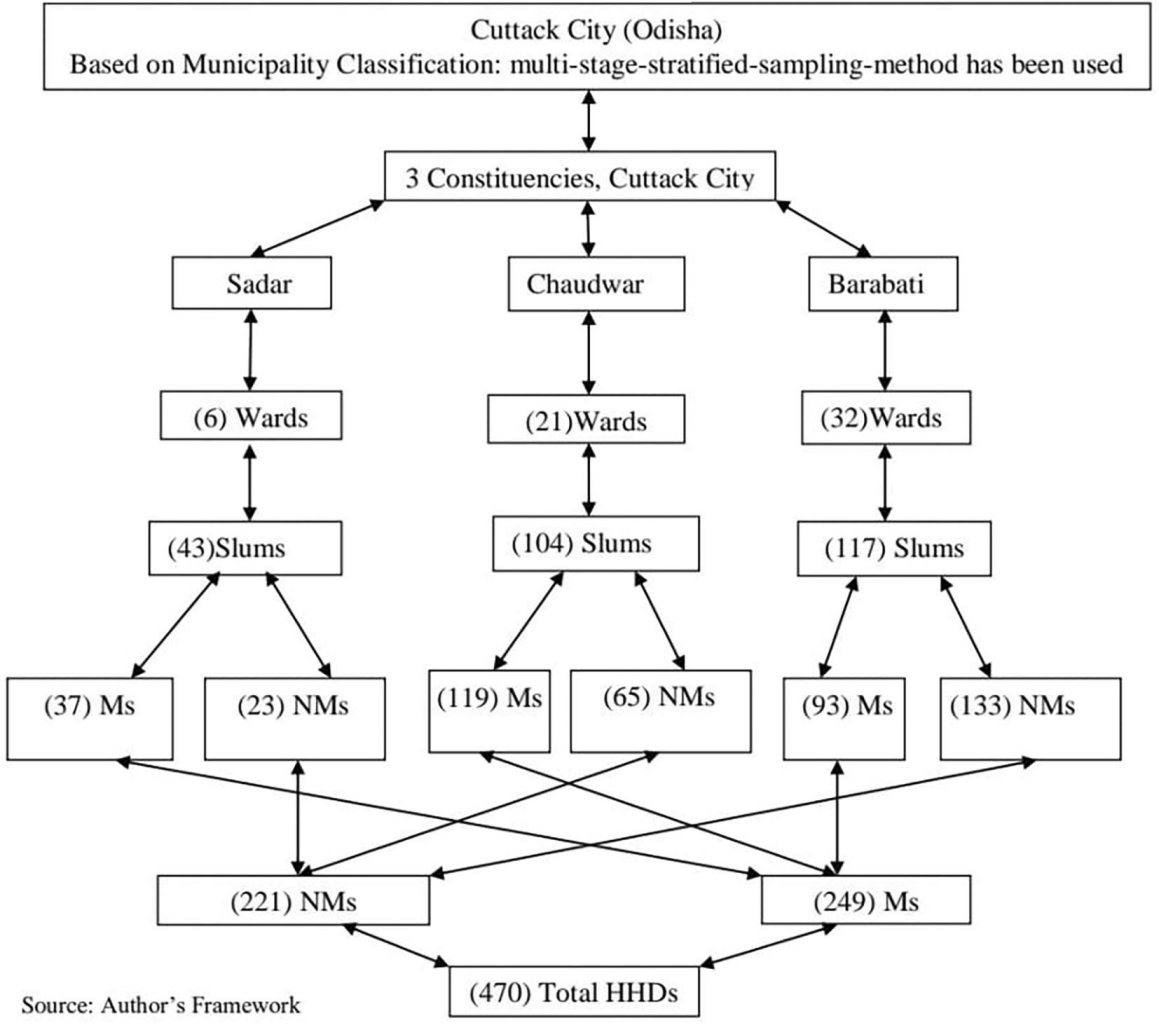
Methodological ways to select the sample.

However, the article is related to the socio-economic problems of informal workers and most of them are illiterate, written consent seems to be impossible. Hence, we preferred to take informed verbal consent. In this regard, we explained the purpose and the content of this research (interview) to the participant, and s/he agreed to participate in the study. Thus, in this study, informed verbal consent was obtained before data collection. Similarly, this research is based on primary survey of the households and specifically to the household head, thus, no minor participants were taken into considered. The methodological steps and the total number of non-migrant and migrant respondents are explained in Figure 1.

## Results and discussion

### Socio-economic summary

The social and economic summary of household heads (HHds), specifically informal workers, was classified into five groups. HHds are classified on the basis of their gender (female or male), marital status (married, unmarried, widowed, separated, or divorced), religion (Muslim or Hindu), social group (General, OBC, SC, or ST), general education level (primary, upper-primary, secondary, higher-secondary, graduate, and above or illiterate), employment (wage-employed/self-employed or casual or contract or unpaid laborer), land category they lived (own/private land or government land) and all categories are explained on the basis of migration status (i.e., either non-migrate or migrated) of the informal workers. The socio-economic summary of the HHds is presented in Table 1, and the values are expressed as percentages.

**Table 1.** Socio-economic summary of the Informal Workers (IWs).

Indicator		Whether the IWs Migrated?		Total (in percent)
		Yes (in percent)	No (in percent)	
Gender	Female	52.9	47.1	22.1
	Male	53.0	47.0	77.9
Marital Status	Unmarried	40.2	59.8	20.6
	Married	55.9	44.1	68.9
	Widowed	53.3	46.7	6.4
	Separated	100	00	0.2
	Divorcee	66.7	33.3	3.8
Religion	Muslim	51.6	48.4	13.2
	Hindu	53.2	46.8	86.8
Social Group	General	64.0	36.0	34.9
	OBC	65.3	34.7	20.2
	SC	40.6	59.4	30.4
	ST	35.3	64.7	14.5
General Education Level	Primary	49.2	50.8	25.5
	Upper Primary	55.2	44.8	14.3
	Secondary	55.4	44.6	25.7
	Higher- Secondary	69.2	30.8	2.8
	Graduate and Above	50.0	50.0	1.7
	Illiterate	51.8	48.2	30.0
Employment Type	Regular Wage Employed	43.9	56.1	31.5
	Self-Employed	50.4	49.6	29.1
	Casual Laborer	62.1	37.9	37.0
	Contract laborer	85.7	14.3	1.5
	Unpaid laborer	25.0	75.0	0.9
Category of land They Lived	Own/Private Land	45.3	54.7	70.0
	Government Land	70.9	29.1	30.0
Total		53.0 (249)	47.0 (221)	100 (470)

Source: From the Field Survey Information.

The social and economic profile of the household head (HHds) helps us understand the socio-economic background of informal workers in the city of 470 HHds, 78 percent (366) were male and 22 percent (104) were female. Among the migrant HHds (53%), 53 percent (194) were male migrant and 52.9 percent (55) were female. Conversely, among non-migrant HHds working in the informal sector, 47 percent (172) were male and 47.1 percent (49) were female workers. This means that informal female workers are lower than male workers in both migrant and non-migrant cases due to lack of safety and security, poor housing conditions, and other essential facilities. Based on marital status, married migrant workers were higher (55.9%) than non-migrant workers (44.1%) due to urgency, financial support, and other responsibilities. Similarly, the percentage of total married informal workers (68.9%) is higher than that of unmarried (20.6), widowed (6.4%), separated (0.2%), and divorced (3.8) informal workers in the informal sector due to more freedom, more responsibility, and no more social restrictions on them.

Similarly, this study found that in the Cuttack City of Odisha there are mostly two religious groups or categories, (Hindu population sample is 86.8 percent or 408 HHds and Muslims are13.2 percent or 62 HHds, respectively), and who are basically engaged in informal sector work, other than all other religious groups. In the case of both the religious groups (Hindu and Muslims), the migrated samples are higher (i.e., 53.2% and 51.6%) than the non-migrated HHds, but in both cases the Hindu migrated HHds are higher than the Muslim migrated HHds and in case of non-migrated samples, Muslim religious HHds are higher than Hindu religious HHds in the Cuttack City of Odisha. This may mean that Muslim religious groups prefer to migrate less than Hindu religious groups (or it may be other religious groups in India, if the samples are available from other migrated religious groups).

Among the various social groups, including migrant and non-migrant HHds, 30 percent (143) were from the SC, 15 percent (68) from the ST, 20 percent (95) from the OBC, and 35 percent (164) from the general category, working in the informal sector. However, in the informal sector general category people are participating in the highest number which is 35 percent then the SC category the 2 ^nd^ highest i.e., 30 percent, and the OBC category (20%) and the ST category is the lowest i.e., 15 percent from the total population of samples. This is only because of lack of information, caste-based demand and the existence of untouchability for the SC and ST categories people, but everything becomes reverse for the general and then for the OBC category people.

On the other hand, the level of general education explained that illiterates are 35 percent, primary education level is 26 percent, upper primary 14 percent, secondary 26 percent, higher secondary is 3 percent, and graduates and above are about 2 percent only. This mean that the general education level of the people is not able to influence the socio-economic conditions of either migrant or non-migrant workers, as education is not considered an indicator to enter into the informal sector in Cuttack City, Odisha.

On the basis of employment, 29 percent (137) are self-employed, 37 percent (174) are casual laborers, 31 percent (148) are regular wage employed, 2 percent (148) are contract laborer and only one percent are HHd unpaid laborers. In between the migrated HHds, 86 percent are contract laborers, 62 percent are casual laborers, 50.4 percent are self-employed, 44 percent are regular wage employed, and only 25 percent are household unpaid laborers. On the other hand, among the non-migrant HHds, 14 percent were contract laborers, 38 percent were casual laborers, 49.6 percent were self-employed, 56 percent were regular wage employed, and 75 percent were unpaid household laborers working in the informal sector of the Cuttack City. However, the study found that among the M informal workers, casual and contract laborers are the highest, and among the non-migrant informal workers, regular wage employed and unpaid laborers are the highest. However, the over all observation of this case study showed that informal M workers are forced to prefer the work as casual labor and informal NM workers are mostly worked as regular wage earners as they are staying there since before and a good network among them, but in both cases, informal workers are generally found to work as casual laborers.

Similarly, on the basis of the category of land they lived, 53 percent had migrated and 47 percent had non-migrated HHds, out of which 30 percent (141) lived on the government land, and 70 percent (329) of HHds are living either on private land or self-owned land. However, among migrant HHds, 71 percent live on the government land, and 45 percent live on private or owned land. On the other hand, among non-migrant HHds (329), 29 percent lived on the govt. land, and 55 percent is on private or self-owned land. Thus, the observation explained that the migrant informal workers prefer and are forced to live on the government land and non-migrant informal workers prefer to live on either private or self-owned land, but both of them (migrants or non-migrants) are mostly prefer to live on the government land as they want to avoid rent. Otherwise, informal workers are generally trying to give a very low amount to the government or to local Dalal (local leader), which would be possible, rather than going to a rented house.

### Migrant and non-migrant informal workers and income variation

To understand the income variation among the NM and M informal workers the study has collected and examined the monthly income of the household head and the result of the descriptive statistics is presented in Table 2. Similar, to the existence of income variation between the groups and among the groups, the study employed the ANOVA analysis which is demonstrated in Table 3.

**Table 2.** Income of informal non-migrant and migrant workers.

Descriptive statistics: (N = 469)				
Status of migration	Income of informal workers			
	Maximum	Minimum	Mean	Std. deviation
Income Before Migration	60,000.00	0.00	1272.07	3448.66
Income After Migration	18,000.00	0.00	3278.26	3680.76

Source: Estimated from the Field Survey Information.

**Table 3.** Group-wise variation in income of the informal workers (ANOVA analysis).

Informal workers’ present income					
Variables name	Sum of squares	df	Mean square	F	Sig.
Category of land They Lived					
Between Groups	78906766.0	1	78906766.0	5.991	.015
Within Groups	6163751701.8	468	13170409.6		
Gender					
Between Groups	645338763.2	1	645338763.2	53.958	.000
Within Groups	5597319704.6	468	11960084.8		
Various Age-groups Household Head					
Between Groups	202186162.4	7	28883737.5	2.209	.032
Within Groups	6040472305.4	462	13074615.4		
Migration Status					
Between Groups	199024065.3	1	199024065.3	15.412	.000
Within Groups	6043634402.5	468	12913748.7		
Availability of Off-Season Supports					
Between Groups	187771988.8	1	187771988.8	14.513	.000
Within Groups	6054886479.0	468	12937791.6		
Type of Employment					
Between Groups	464824729.4	4	116206182.4	9.352	.000
Within Groups	5777833738.4	465	12425448.9		
Social Groups					
Between Groups	163157663.697	3	54385887.899	4.169	.006
Within Groups	6079500804.1	466	13046139.1		
Religions					
Between Groups	3034797.3	1	3034797.3	.228	.634
Within Groups	6239623670.5	468	13332529.2		
Principal Sectors					
Between Groups	636817391.2	9	70757487.9	5.806	.000
Within Groups	5605841076.6	460	12186611.0		
Number of Dependency					
Between Groups	403436855.9	8	50429607.0	3.981	.000
Within Groups	5839221611.9	461	12666424.3		
Marital Status					
Between Groups	329285635.8	4	82321408.9	6.473	.000
Within Groups	5913372832.0	465	12716930.8		
General Education Level					
Between Groups	206853508.2	6	34475584.7	2.645	.016
Within Groups	6035804959.6	463	13036295.8		
Eligible for Paid Leave					
Between Groups	2595716.1	1	2595716.1	.195	.659
Within Groups	6240062751.7	468	13333467.4		
Availability of Social Security Benefits					
Between Groups	11058710.5	3	3686236.8	.276	.843
Within Groups	6231599757.3	466	13372531.7		
Member of the Union					
Between Groups	302651362.3	2	151325681.2	11.897	.000
Within Groups	5940007105.4	467	12719501.3		
Total	6242658467.8	469			

Source: ANOVA Analysis Estimated from the Field Survey Information.

The descriptive statistics in Table 2 show that there is a significant difference in income before and after migration. After migration, the average monthly income of the migrant informal workers is higher than that before the migration, that is, the average monthly income is Rs. 3,278.26 and Rs. 1,272.07, respectively. Similarly, it is also the same in the case of the maximum income and the standard deviation of the informal migrant workers, that is, Rs. 60,000.00 and Rs. 3448.66, are higher than the Rs. 18,000.00 and Rs. 3680.76, respectively. However, the pathetic situation is that all the informal workers (NM and M) are facing the ‘situation of zero minimum income’ due to unavailability of work in all the seasons, months, and days in the city.

### Informal workers and group-wise income variations

ANOVA analysis was used to understand whether the variation in income existed (between the groups and among the groups). In this regard, H _0_ and H _1_ are the null and alternative hypotheses, respectively, considering (where H _o_ implies the existence of variations in mean income, which is equal for all groups, and H _1_ implies variations in mean income, which is not equal for all groups). In this study, the null-hypothesis for the category of land they lived (government and private/self-owned land), gender (male and female), various age groups of household head, migration status (migrated or not migrated), availability of off-season support (yes or no), employment types (unpaid laborer, contract laborer, casual laborer, wage employed, and self-employed), social groups (General, OBC, SC or ST), principal sectors, number of dependencies, marital status (married, unmarried, widowed, divorced or separated), general education level (illiterate, primary schooling, upper primary schooling, secondary, higher secondary and graduate and above), and members of the union (yes, no, and not aware) have turned to be significant as the P-values are less than 0.05. This indicates the existence of mean income differences among and within the groups in the informal sector. Therefore, we reject the null hypothesis of these significant variables and conclude that different groups have different mean incomes per month. However, variables such as religion (Hindu or Muslim), eligibility for paid leave (yes or no), and availability of social security benefits (providing only health care, only maternal benefits and only gratuity, only PF/pension, not eligible or not known) were not significant, as the P-values of these variables were greater than 0.05. Thus, rejection of the null hypothesis is not possible for these variables and it can be concluded that there is no variation in the mean income of the different groups of informal-sector workers.

### Determinants of income of informal workers (Migrant and Non-migrants)

To determine the determinants of the income of informal sector workers at the regional level, this study applied regression analysis followed by ANOVA. The analysis was also applied separately for migrant and non-migrant informal workers to identify the differences between these two groups of informal workers. Thus, to determine the important determinants of informal income, the multiple regression equation is formulated (where Y is the monthly informal income, β _j_ is the = coefficient of independent variable, and j = 1, 2, 3, 4, …, 18) as:

$$Y=\text{Constant}+\text{Casual Labor}\;\left(\beta_{1}\right)+\text{Regular Wage Employed}\left(\beta_{2}\right)+\mathrm{SC}\left(\beta_{3}\right)+\mathrm{ST}\left(\beta_{4}\right)+\text{General}\left(\beta_{5}\right)+\text{Dependant}\left(\beta_{6}\right)+\text{General Education Level}\;\left(\beta_{7}\right)+\text{Vocational Training}\;\left(\beta_{8}\right)+\mathrm{Off}\;\text{Season Support}\;\left(\beta_{9}\right)+\text{Gender}\;\left(\beta_{10}\right)+\text{Union}\;\left(\beta_{11}\right)+\mathrm{Age}\;\text{of HHHs}\;\left(\beta_{12}\right)+\mathrm{Age}\;\text{Squared of HHHs}\;\left(\beta_{13}\right)+\text{Land Owned}\;\left(\beta_{14}\right)+\text{Migration}\;\left(\beta_{15}\right)+\text{Services Sector}\;\left(\beta_{16}\right)+\text{Agriculture Sector}\;\left(\beta_{17}\right)+\text{Household Sector}\;\left(\beta_{18}\right)\ldots$$ (1)

Similarly, to determine the effective and significant determinants of the migrant and non-migrant informal workers, multiple regression models were formulated separately as follows:

$$Y=\text{Constant}+\text{Casual Labor}\;\left(\beta_{1}\right)+\text{Regular Wage Employed}\;\left(\beta_{2}\right)+\mathrm{SC}\;\left(\beta_{3}\right)+\mathrm{ST}\;\left(\beta_{4}\right)+\text{General}\;\left(\beta_{5}\right)+\text{Dependant}\left(\beta_{6}\right)+\text{General Education Level}\;\left(\beta_{7}\right)+\text{Vocational Training}\;\left(\beta_{8}\right)+\mathrm{Off}\;\text{Season Support}\;\left(\beta_{9}\right)+\text{Gender}\left(\beta_{10}\right)+\text{Union}\;\left(\beta_{11}\right)+\mathrm{Age}\;\text{of HHHs}\;\left(\beta_{12}\right)+\mathrm{Age}\;\text{Square of HHHs}\;\left(\beta_{13}\right)+\text{Land Owned}\;\left(\beta_{14}\right)+\text{Services Sector}\;\left(\beta_{15}\right)+\text{Agriculture Sector}\;\left(\beta_{16}\right)+\text{Household Sector}\;\left(\beta_{17}\right),\text{for}\;i=1,2\ldots$$ (2)

Where, ‘Yi’ is monthly income of migrant informal workers (i = 1) and monthly income of non-migrant informal workers (i = 2), ‘β _j_’ = independent variables coefficients, and j = 1, 2, 3, … 17. However, all variables are dummy variables in these models (except age, age squared, and land owned). In this regard, Equations (1) and (2) are estimated using the OLS method, and the results of Equations (1) and (2) are presented in Tables 4, 5, and 6, respectively.

**Table 4.** Determinants of income (Informal workers).

Dependant Variable (Y): Monthly Income				
Variables	Coef. (β)	Std. Err.	t	Sig. Level (P)
Casual Labor	-931.02	367.56	-2.53	0.012 **
Regular Wage Employed	-1142.26	393.84	-2.9	0.004 *
SC	-762.86	445.28	-1.71	0.087
ST	-704.64	531.38	-1.33	0.185
General	-842.89	413.90	-2.04	0.042 **
Dependent	286.70	97.30	2.95	0.003 *
General Education Level	128.16	121.19	1.06	0.291
Vocational Training	798.08	321.22	2.48	0.013 **
Off-season Support	838.28	325.15	2.58	0.01 *
Gender	1321.37	419.61	3.15	0.002 *
Union	-1823.67	484.46	-3.76	0 *
Age of HHHs	167.69	59.44	2.82	0.005 *
Age Square of HHHs	-2.18	0.70	-3.12	0.002 *
Land Owned	-12.19	4.40	-2.77	0.006 *
Migration	1455.30	404.18	3.6	0 *
Services Sector	-1669.50	511.74	-3.26	0.001 *
Agriculture Sector	-1939.31	689.35	-2.81	0.005 *
Household Sector	-1258.44	469.09	-2.68	0.008 *
Constant	3930.45	1716.21	2.29	0.022 **
R-squared	0.295			
Adj R-squared	0.267			
Prob > F	0			
Root MSE	3124			
F (18, 451)	10.48			
Observations (total)	470			

Source: Estimated from the Field Survey Information.

* Implies a 1% or Less than 1% significant level;

** Implies a 5% significance level.

**Table 5.** Determinants of income (Only migrant workers).

Monthly Income: Dependant Variable (Y)				
Variables	Coef. (β)	Std. Err.	t	Sig. Level (P)
Casual Laborer	-194.51	404.95	-0.48	0.631
Regular Wage Employed	-1269.50	460.24	-2.76	0.006 *
SC	-134.52	498.84	-0.27	0.788
ST	-887.80	634.43	-1.4	0.163
General	-138.41	413.12	-0.34	0.738
Dependents	198.27	121.07	1.64	0.103
General Education Level	184.31	125.09	1.47	0.142
Vocational Training	285.70	366.37	0.78	0.436
Off-season Support	1254.29	340.01	3.69	0 *
Gender	1641.32	483.29	3.4	0.001 *
Union	129.17	540.72	0.24	0.811
Age of the HHHs	146.50	71.49	2.05	0.042 **
Age Square of the HHHs	-2.12	0.88	-2.42	0.016 **
Land Owned	-18.70	7.55	-2.47	0.014 **
Services Sector	-783.73	588.04	-1.33	0.184
Agriculture Sector	-47.07	1107.53	-0.04	0.966
Household Sector	-1327.80	557.42	-2.38	0.018 **
Constant	1687.80	2053.56	0.82	0.412
R-squared	0.34			
Adj R-squared	0.30			
Prob > F	0			
Root MSE	2482.9			
F(17, 231)	7.11			
Observations (only migrants)	249			

Source: Estimated from the Field Survey Information.

* Implies a 1% or Less than 1% significant level;

** Implies a 5% significance level.

**Table 6.** Determinants of income (Only non-migrant workers).

Monthly Income: Dependant Variable (Y)				
Variables	Coef. (β)	Std. Err.	t	Sig. Level (P)
Casual Laborer	-1579.60	641.00	-2.46	0.015 **
Regular Wage Employed	-986.74	636.42	-1.55	0.123
SC	-1484.40	801.55	-1.85	0.065
ST	-980.59	915.80	-1.07	0.286
General	-1869.10	829.67	-2.25	0.025 **
Dependents	272.18	151.31	1.8	0.074
General Education Level	124.82	244.72	0.51	0.611
Vocational Training	1264.04	573.11	2.21	0.029 *
Off-season Support	576.49	609.99	0.95	0.346
Gender	1294.17	733.25	1.76	0.079
Union	-3555.60	825.98	-4.3	0 *
Age of the HHHs	201.06	99.26	2.03	0.044 **
Age Square of the HHHs	-2.50	1.11	-2.24	0.026 **
Land Owned	-6.97	6.33	-1.1	0.272
Services Sector	-2198.30	848.39	-2.59	0.01 *
Agriculture Sector	-2652.30	957.54	-2.77	0.006 *
Household Sector	-937.85	762.95	-1.23	0.22
Constant	7374.42	3065.57	2.41	0.017 **
R-squared	0.31			
Adj R-squared	0.26			
Prob > F	0			
Root MSE	3621.3			
F (17, 203)	5.44			
Observations (only non-migrants)	221			

Source: Estimated from the Field Survey Information.

* Implies a 1% or Less than 1% significant level;

** Implies a 5% significance level.

In the regression model, as explained by the independent variables, the estimated R-square is 0.2949 which shows that there is only 26 percent of the income variation in ISWs. In the midst of independent variables, the regular wage employed, dependants on the HHds, off-season support to the HHds, gender of the HHds, availability of union for the informal sector workers, age and age square of the HHds, land owned, migration status, service sector, agricultural sector, and household sector are the significant variables influencing at the 1 percent or less than 1 percent significance level. Similarly, casual laborers, general category people, and the availability of vocational training are the momentous variables that influence the income of informal sector workers at the 5% significance level. However, among the independent variables, the number of dependents on the HHds, vocational training of the HHds, off-season support, gender, age of the HHds, and migration status of the ISWs had a positive and significant influence. Overall, socio economic variables played a vital role in determining the monthly income of this informal group.

Equation (2) is estimated for migrant workers using the ordinary least squares (OLS) method, and the results are shown in Table 5.

The regression model only for the income of the migrant workers, the estimated R-square of 0.344, explains that there is only 34 percent of the income variation of the ISWs. In the midst of independent variables, the regular wage employed, off-season support to the HHds, gender of the HHds, and the availability of unions for informal sector workers were found to be significant variables at the 1 percent or less than 1 percent significance level. Similarly, the age and age squared of the HHds, land owned and the household sector have been significant variables influencing the income of the informal sector workers at the 5% significance level. However, regular workers, land owned by HHds, and age squared negatively affect the income of M workers. Conversely, off-season support, gender of the HHds and age of the HHds significantly and positively influenced informal migrant workers only.

Similarly, Equation (2) is estimated for non-migrant workers using the OLS method, and the results are shown in Table 6.

The regression model only for the income of the non-migrant workers, the estimated R-square of 0.313, explains that there is only 31 percent of the income variation of the informal sector workers. In the midst of independent variables, the availability of vocational training and, labor unions for the informal sector workers, service sector, and agricultural sector seem to have significant variables at the 1% or less than 1% significance level. Similarly, casual laborers, general category people of society, age, and age square of the HHds were found to be significant variables influencing the income of informal sector workers at the 5% significance level. However, the casual laborer, general category people, labor union, age square of the HHds, service sector, and agricultural sector negatively affect informal migrant workers’ incomes. On the other hand, vocational training centers (received either through special training centre or through their hereditary system) and the age of the HHds are positively significant to the income of non-migrant informal workers.

## Conclusions and policy implications

After a ground-level data collection and analysis, the study may be concluded by slating that informal sector workers, both Ms and NMs, have greater attention at both the national and regional levels for their upliftment in modern society. The findings of this study also show that even though the income of non-migrants is lower than that of migrants due to lower paid wage earnings (e.g., security guard jobs, working as a cook or maid in the household sector, and local rickshaw puller), other livelihood facilities such as local unity, social and job security, sanitation, and other facilities are enjoyed by them due to their voting rights. All the informal workers, both M and NM, are facing the ‘situation of zero minimum income’ due to unavailability of work in all the seasons-months-days in the city. Thus, various socio-economic factors such as casual laborers, regular wage employed, general category people, number of dependencies, vocational training, off-season supporters, gender, memberships with the union, age, age squared, land owned by the HHds, migration status, service sector, agriculture sector, and household sector play a crucial role in these lower income groups and variations.

However, none of these determinants are constant for non-migrant and migrant informal workers in the city. Over all, informal workers are the ultimate victims in cities. Thus, in this post COVID-19 pandemic situation, there is a need for improvements in the area of basic facilities such as housing, electricity, health, vocational education, and social security through *citizen forum* (a system of open forum in the Bhubaneswar municipality for discussing the problems is faced by public utility and also used as a platform for providing possible solutions to the problem).

The present informal employment study, therefore, needs paramount importance for the country, state and district or from the grass root level (villages) to central government (following the ‘Panchayat Raj System’ in India, i.e. a three-tier development structure for the rural development), 1stly And 2ndly, to develop the poor socio-economic conditions of informal sector workers, both rural and urban governments should facilitate basic amenities such as sanitary facilities, proper drinking water facilities, basic nutritional food facilities at minimal cost, vocational education or training, and regulated shelter facilities with minimal or free costs. In other words, in a traditional sense we can say that we can provide and facilitate the “Roti-Kapda-Makaan” to these vulnerable sections of the state and country. 3rdly, for creating the awareness about health and environment, both the local and central government should facilitate the basic education through “M-E-S” (Mobile-Education-Service, which means, education and awareness services provided by the vehicles to their door step just such as the venders selling their goods and services on everyday basis) to these informal sector workers. However, 4thly, the most important policies need to be re-emphasized by the government—the MGNREGS (Mahatma Gandhi National Rural Employment Guarantee Schemes) and MSMEs (Micro, Small, and Medium Enterprises) — which can play a dual role in reducing unemployment and increasing income both in the village and the urban areas of the country in this post COVID-19, war and inflationary situation.

Therefore, by implementing and applying the above policies for the upliftment of the informal sector workers can also be able to achieve many Sustainable Goals, such as 1) No Poverty (by giving the employment opportunities both in the urban and rural areas), 2) Zero Hunger (by making available the basic nutritional food at minimal cost), 3) Good Health and Well-Being (by creating the awareness about the health and environment), 4) Quality Education (by facilitating the basic education and vocational training through new ‘M-E-S’ programme), 6) Clean Water and Sanitation (by facilitating the basic amenities such as sanitary facilities, proper drinking water facilities), 8) Decent Work and Economic Growth (by generating the work in the organized sector), 9) Industry, Innovation and Infrastructure (by re-emphasizing on the MGNREGS and MSME programmes), 12) Responsible Consumption and Production (by providing the basic nutritional food at minimal cost or minimum support price), and 17) Partnerships for the Goals (by the involvement of both the informal sector workers and the state and central government for achieving all these goals). However, this primary research work is also not free from limitations due to its limited sample size (470 only), restricted area (Cuttack District, Odisha only), lack of information before their migration, and the situation after the COVID-19, war, and hyper inflation. This study is also applicable to developing countries such as India, where the number of informal sectors (more than 90%) and workers (more than 91%) are large.

## Declarations

Ethical Clearance: This study meets national and international guidelines for research on humans. In this regard, an ethical approval certificate has been obtained by University level Ethics Committee (ULEC), KIIT Deemed to be University, Bhubaneswar with the ethical clearance certificate no. KIIT/ULEC/012/2025 on dated 15/05/2025.

Materials Used: Due to the primary nature of the article, we have prepared a structured questionnaire for the respondents for data analysis (The Figshare DIO/Identifier no 10.6084/m9.figshare.30581063).

## Data Availability

Figshare, Data for F1000Research, https://doi.org/10.6084/m9.figshare.30581063.v1. Barik, S. (2025). This project contains the following underlying data:
• Questionnaire • Field data Data are available under the terms of the Creative Commons Attribution 4.0 International license (CC-BY 4.0).

## Footnotes

^1^
According to NCEUS 2007, unincorporated private enterprises with less than 10 workers engaged in production of goods and services on a proprietary basis and provide the employment without covering the social security benefits to the workers.
