# Supplementary material for: Socio-economic Conditions & Determinants of Income: A Tale of Informal Workers of Urban Odisha, India
Source: F1000Res. 2026 May 26;15:253. Originally published 2026 Feb 13. [Version 2] doi: 10.12688/f1000research.168208.2 (PMC13270184; doi:10.12688/f1000research.168208.2)
Supplement: Peer review history

# Reviewer report: Reviewer response for version 2

**DOI:** 10.5256/f1000research.201058.r489192

**Published:** 2026-06-15

**Authors:** Deepak Kumar Behera

**Affiliations:** 1 National Institute of Technology Patna, Patna, Bihar, India

This study examines the socioeconomic conditions and determinants of income among informal sector workers in Cuttack city, Odisha, with a particular focus on migrant and non-migrant workers. Using primary data collected through a multi-stage stratified sampling method and analyzed through ANOVA and multiple regression techniques, the study identifies key factors influencing income levels in the informal sector.

 The findings reveal that migrant workers generally earn higher incomes than non-migrant workers, mainly because they are engaged in occupations with better wage structures and payment patterns. In contrast, non-migrant workers are predominantly involved in low-paid household services. Several socioeconomic factors significantly affect workers’ income, including housing conditions, gender, age of the household head, migration status, access to off-season employment support, type of employment, social group, religion, sector of work, dependency ratio, and union membership.

 The study concludes that improving access to basic amenities and services such as housing, electricity, healthcare, vocational education, social security, and employment opportunities is essential for enhancing the welfare of informal workers. It recommends strengthening mobile education services, expanding MGNREGS initiatives, and promoting the MSME sector to support sustainable and inclusive development while contributing to the achievement of multiple Sustainable Development Goals

 The methodology section lacks sufficient details regarding the sampling design.

 Operational definitions of variables such as migration status, informal income, off-season support, dependency ratio, and employment type should be provided.

Is the work clearly and accurately presented and does it cite the current literature?

Yes

If applicable, is the statistical analysis and its interpretation appropriate?

Yes

Are all the source data underlying the results available to ensure full reproducibility?

Yes

Is the study design appropriate and is the work technically sound?

Yes

Are the conclusions drawn adequately supported by the results?

Yes

Are sufficient details of methods and analysis provided to allow replication by others?

Partly

Reviewer Expertise:

Labour and Employment, Development Studies

I confirm that I have read this submission and believe that I have an appropriate level of expertise to confirm that it is of an acceptable scientific standard.

# Reviewer report: Reviewer response for version 2

**DOI:** 10.5256/f1000research.201058.r489193

**Published:** 2026-06-13

**Authors:** Jajati Keshari Parida

**Affiliations:** 1 School of Economics, University of Hyderabad, Hyderabad, Telangana, India

1. The Y-variable should be renamed 'earnings' instead of 'income'. This earnings equation should be estimated using the Heckman selection model or a Tobit regression rather than an OLS model. This causes estimation bias.

 2. The earning difference (differentials) between migrant and non-migrant workers can be estimated with some statistical significance. At least it should be supported by a t-test.

 3. Please check it for language editing. A few typos are still there.

 I wish the Authors all the best. Hope these comments would help improve this paper.

Is the work clearly and accurately presented and does it cite the current literature?

Yes

If applicable, is the statistical analysis and its interpretation appropriate?

Partly

Are all the source data underlying the results available to ensure full reproducibility?

Yes

Is the study design appropriate and is the work technically sound?

Partly

Are the conclusions drawn adequately supported by the results?

Yes

Are sufficient details of methods and analysis provided to allow replication by others?

Partly

Reviewer Expertise:

Employment, Migration and Poverty

I confirm that I have read this submission and believe that I have an appropriate level of expertise to confirm that it is of an acceptable scientific standard.

# Reviewer report: Reviewer response for version 1

**DOI:** 10.5256/f1000research.185374.r461830

**Published:** 2026-03-19

**Authors:** Rashidat Sumbola Akande

**Affiliations:** 1 Kwara State University, Malete, Nigeria

The article “Socio-economic Conditions & Determinants of Income: A Tell of Informal Workers of Urban Odisha, India” explores the income dynamics and living conditions of informal workers in Cuttack City, Odisha. The authors collected primary data from 470 household heads using a multi-stage stratified sampling method and analysed the results with ANOVA and regression techniques. Their findings suggest that migrant workers generally earn more than non-migrant workers, largely because they are engaged in casual or contract labour, while non-migrants are more concentrated in household services with lower pay. Key determinants of income include land tenure, gender, age of household head, migration status, employment type, social group, religion, dependency ratio, and union membership. The authors conclude with policy recommendations that emphasize improving housing, health, education, and employment opportunities.

 While the study is timely and relevant, several areas require improvement to make the article scientifically sound. The title, though descriptive, is not well phrased. A clearer version such as “Socio-economic Conditions and Determinants of Income among Informal Workers in Urban Odisha, India” would improve readability.

 The abstract is concise but lacks methodological detail and specific quantitative results, which should be added to give readers a clearer sense of the study’s scope and findings.

 The literature review is extensive but overly descriptive. It cites many international and national studies without synthesizing them into a coherent narrative or clearly identifying the research gap. The authors should explicitly state how their work addresses an existing gap in the literature.

 The methodology section needs stronger justification for the sample size and more detail on ethical safeguards, particularly regarding confidentiality. The choice of ANOVA and regression should be explained more thoroughly and justified.

 The results section provides a rich socio-economic profile but does not adequately interpret the regression analysis. The authors should present coefficients, p-values, and confidence intervals, and discuss the implications of each determinant more explicitly. The tables are informative but require clearer captions and tighter integration into the discussion.

 The conclusion and policy recommendations are broad and somewhat generic. They should be directly tied to the empirical findings.

 Finally, the referencing style is inconsistent and should be revised to conform to the journal’s specification. All in-text citations must correspond to entries in the reference list.

Is the work clearly and accurately presented and does it cite the current literature?

Yes

If applicable, is the statistical analysis and its interpretation appropriate?

Partly

Are all the source data underlying the results available to ensure full reproducibility?

No source data required

Is the study design appropriate and is the work technically sound?

Partly

Are the conclusions drawn adequately supported by the results?

Partly

Are sufficient details of methods and analysis provided to allow replication by others?

Yes

Reviewer Expertise:

Sustainable Development, Informal Economy, Migration

I confirm that I have read this submission and believe that I have an appropriate level of expertise to confirm that it is of an acceptable scientific standard, however I have significant reservations, as outlined above.

# Reviewer report: Reviewer response for version 1

**DOI:** 10.5256/f1000research.185374.r459274

**Published:** 2026-03-18

**Authors:** Eric Amoo Bondzie

**Affiliations:** 1 University of Cape Coast, Cape Coast, Ghana

**Summary**

 The study uses primary data collected via multi‑stage stratified sampling and a structured questionnaire of informal sector workers in Cuttack, Odisha, analysing determinants of informal income with ANOVA and multiple regression. It finds that migrant workers earn more than non‑migrants due to job type and payment patterns and identifies key socioeconomic determinants of lower informal incomes: land category, gender, age of household head, migration status, off‑season support, employment type, social group, religion, sector, dependents, and union membership. The authors recommend policy actions to improve housing, utilities, health, vocational education, social security, and employment, highlighting mobile education services, MGNREGS, and MSME support as pathways to advance multiple Sustainable Development Goals.

**Introduction and objectives:**

1. The topic should be "Tale" and not "Tell." Check and correct.
2. A primary household survey with multi‑stage stratified sampling and 470 observations is an appropriate empirical base for descriptive and regression analysis of local informal labour markets.
3. The paper should more explicitly discuss external validity limits (single‑city study), potential seasonal bias (survey March to May), and sample representativeness for different informal subsectors.
4. The paper should add a short paragraph quantifying sampling coverage (share of slum population sampled), response rate, and any non‑response or selection issues; discuss how findings may or may not generalize beyond Cuttack.

**Literature reviews:**

1. The paper would benefit from a tighter theoretical framing that links specific hypotheses to empirical tests. For example, state explicit hypotheses such as:

*H1: Migrant status increases income because migrants concentrate in higher‑paid casual construction and contract work; H2: Lack of land tenure reduces bargaining power and lowers income.*

 2. Add recent microeconometric studies on urban informal earnings and migration in India (post‑2018) that use decomposition or quantile regression methods; this will help justify the choice of ANOVA and OLS and suggest alternative estimators.

 3. Expand discussion of local institutions (labour market intermediaries, union presence, MGNREGS linkages) that the recommendations later rely on. **Methodology:**

1. **Model specification and dependent variable**: Clarify the exact dependent variable (monthly income, log income, per capita household income?) and its distribution. If income is skewed, use log transformation or robust estimators.
2. **Regression details missing: **Report full regression tables: coefficient estimates, standard errors, R², sample size, and model diagnostics. Indicate whether standard errors are robust to heteroskedasticity.
3. **Functional form and heterogeneity**: Consider **quantile regression** to capture heterogeneity across the income distribution (effects on low‑income vs higher‑income informal workers). The paper’s policy claims about the “lower income” group would be strengthened by quantile estimates.
4. **Endogeneity and omitted variables**: Migration status may be endogenous (self‑selection). Discuss potential bias and, if possible, implement sensitivity checks (e.g., control for pre‑migration characteristics, duration since migration, or use propensity‑score matching as a robustness check).
5. **Variable construction and measurement**: Provide precise definitions and coding for categorical variables (social group, employment type, off‑season support, union membership). For example, how is “off‑season support” measured?
6. **Multiple hypothesis testing**: Given many covariates, discuss the risk of false positives and consider reporting adjusted p‑values or focusing on a smaller set of pre‑specified determinants.

**Results and discussions**

1. The manuscript sometimes implies causality (e.g., migration *causes* higher income). Rephrase to reflect associational evidence unless selection is addressed.
2. The paper reports that migrants earn more but does not present effect sizes in an interpretable way (e.g., percentage difference in mean or median income). Add clear magnitudes and confidence intervals.
3. The discussion would be stronger if the authors linked income differences to mechanisms: show regressions where employment type mediates the migration–income relationship, or present mediation analysis (e.g., include employment type and observe change in migration coefficient).
4. Present robustness checks: alternative income definitions, excluding outliers, separate regressions for men and women, and tests for multicollinearity among covariates.
5. Improve table clarity: include sample sizes per cell, standard deviations, and p‑values for ANOVA. Add a regression appendix with full model outputs.

**Conclusions and recommendations**

1. Distinguish short‑term, medium‑term, and long‑term policy actions. For example, short‑term land tenure regularisation and affordable housing.
2. Specify which agencies should lead (municipal corporation, state labour department, skill development mission) and suggest pilot designs (e.g., a targeted MGNREGS pilot for migrant slum clusters).
3. Even a qualitative discussion of fiscal and administrative feasibility would help policymakers assess trade‑offs.
4. Propose measurable indicators to track progress (changes in average informal income, share of workers with social security, number of trainees placed in MSMEs).
5. Given the low female participation reported, include targeted measures for women’s safety, childcare, and training to increase female labour force participation and earnings.

Is the work clearly and accurately presented and does it cite the current literature?

No

If applicable, is the statistical analysis and its interpretation appropriate?

Partly

Are all the source data underlying the results available to ensure full reproducibility?

No

Is the study design appropriate and is the work technically sound?

Partly

Are the conclusions drawn adequately supported by the results?

No

Are sufficient details of methods and analysis provided to allow replication by others?

No

Reviewer Expertise:

Macroeconomics, Monetary Economics, DSGE modelling, Public Economics and Time Series Econometrics.

I confirm that I have read this submission and believe that I have an appropriate level of expertise to state that I do not consider it to be of an acceptable scientific standard, for reasons outlined above.
